# Literature on Wearable Technology for Connected Health: Scoping Review of Research Trends, Advances, and Barriers

**DOI:** 10.2196/14017

**Published:** 2019-09-05

**Authors:** Tatjana Loncar-Turukalo, Eftim Zdravevski, José Machado da Silva, Ioanna Chouvarda, Vladimir Trajkovik

**Affiliations:** 1 Faculty of Technical Sciences University of Novi Sad Novi Sad Serbia; 2 Faculty of Computer Science and Engineering Saints Cyril and Methodius University Skopje North Macedonia; 3 Institute for Systems and Computer Engineering, Technology and Science Faculty of Engineering University of Porto Porto Portugal; 4 Faculty of Health Sciences Aristotle University of Thessaloniki Thessaloniki Greece

**Keywords:** wearable technology, telemedicine, assisted living facilities, review

## Abstract

**Background:**

Wearable sensing and information and communication technologies are key enablers driving the transformation of health care delivery toward a new model of connected health (CH) care. The advances in wearable technologies in the last decade are evidenced in a plethora of original articles, patent documentation, and focused systematic reviews. Although technological innovations continuously respond to emerging challenges and technology availability further supports the evolution of CH solutions, the widespread adoption of wearables remains hindered.

**Objective:**

This study aimed to scope the scientific literature in the field of pervasive wearable health monitoring in the time interval from January 2010 to February 2019 with respect to four important pillars: technology, safety and security, prescriptive insight, and user-related concerns. The purpose of this study was multifold: identification of (1) trends and milestones that have driven research in wearable technology in the last decade, (2) concerns and barriers from technology and user perspective, and (3) trends in the research literature addressing these issues.

**Methods:**

This study followed the scoping review methodology to identify and process the available literature. As the scope surpasses the possibilities of manual search, we relied on the natural language processing tool kit to ensure an efficient and exhaustive search of the literature corpus in three large digital libraries: Institute of Electrical and Electronics Engineers, PubMed, and Springer. The search was based on the keywords and properties to be found in articles using the search engines of the digital libraries.

**Results:**

The annual number of publications in all segments of research on wearable technology shows an increasing trend from 2010 to February 2019. The technology-related topics dominated in the number of contributions, followed by research on information delivery, safety, and security, whereas user-related concerns were the topic least addressed. The literature corpus evidences milestones in sensor technology (miniaturization and placement), communication architectures and fifth generation (5G) cellular network technology, data analytics, and evolution of cloud and edge computing architectures. The research lag in battery technology makes energy efficiency a relevant consideration in the design of both sensors and network architectures with computational offloading. The most addressed user-related concerns were (technology) acceptance and privacy, whereas research gaps indicate that more efforts should be invested into formalizing clear use cases with timely and valuable feedback and prescriptive recommendations.

**Conclusions:**

This study confirms that applications of wearable technology in the CH domain are becoming mature and established as a scientific domain. The current research should bring progress to sustainable delivery of valuable recommendations, enforcement of privacy by design, energy-efficient pervasive sensing, seamless monitoring, and low-latency 5G communications. To complement technology achievements, future work involving all stakeholders providing research evidence on improved care pathways and cost-effectiveness of the CH model is needed.

## Introduction

### Background

As the worldwide population grows and the access to health care is increasingly being demanded, real-time monitoring of various physiological signals has driven the research and development of diverse wearable and implantable systems. Connected health (CH) describes the new paradigm of a technology-enabled model of health and lifestyle management [1]. It is implicitly a multidisciplinary technology domain set up to provide preventive and remote treatments. CH relies on a digital information structure based on the internet, sensing, communications, and intelligent techniques, in support of health-related applications, systems, and engineering.

Wearables, as well as hearables (in-ear devices) and nearables (neighboring devices that interact with wearables) integrated into the wider concept of Internet of Things (IoT), are being considered the most likely technologies to transform future health care and lifestyles [2,3]. This revolution began with the smartphone, which is now becoming a widespread intrusive and ubiquitous technology. Most current wearables and nearables are equipped with different types of sophisticated sensors. Different types of sensors powered by advanced analytics are being explored to develop functionalities of truly portable medical laboratories. Seamless integration of these measurements in smartphone apps permits for targeted information to be delivered on time, enhancing the user experience in typical assisted living scenarios. The general acceptance, ease of use, and reliability of smartphones facilitates user adherence to different added value apps that allow filling a gap in the area of self-physiological sensing and fitness monitoring [4]. Wearable technology has become mainstream, with the most significant influence on fitness and health care industries [2].

The importance gained by wearables among consumer devices can be tracked by their increasing share in consumer electronics shows promoting self-care and health management. According to the International Data Corporation, 172.2 million wearable units were shipped in 2018 [4], and this number is expected to grow, contributing significantly to the revolution of the IoT market [5]. Advances in wearable technologies and user acceptance of available consumer wearable devices pave the pathway toward seamless physiological monitoring.

The first body area networks (BANs) and wearable units comprised a number of sensors with a processing unit and wireless nodes assembled on printed circuit boards [6,7]. The design was bulky and uncomfortable, accompanied by large batteries, and had numerous issues associated with frequent recharge and loss of data communication. Since then, tremendous progress has been made in sensing technologies. The bulky design is being rescaled to a system on chip. Lowered power consumption, reliable communications, distributed processing, and data analytics improved the potential of wearables and made a significant impact on technology acceptance [7]. The technology innovations directly responded to user-related concerns (sensor miniaturization, seamless monitoring, secured communications, lower power consumption, energy harvesting, and plug-and-play functionalities) as well as safety and security (reliable sensing and data preprocessing, secured data communication, and reliable analytics).

However, the user feedback reviews report that initial user enthusiasm on wearables is often lost because of unclear use cases (unclear end user need), price, and associated complexities in device pairing with a smartphone [8]. The translation to long-term commitment to wearables requires clear use scenarios, valuable feedback, and constructive recommendations [8,9]. The inevitable transformation from a traditional, reactive health care model to a proactive and preventive model will bring clear use cases of CH solutions for early diagnostic or chronic condition monitoring [1]. Innovative CH scenarios are strongly motivated, exact, and economically beneficial [3,10].

The role that sensing, and information and communication technologies have gained as essentials in digital health has been summarized and elaborated in numerous research articles on sensors, data analytics, and secure and reliable communication platforms for CH solutions [3,10-16]. To stimulate and facilitate knowledge transfer and dissemination among policymakers and stakeholders, it is equally important to summarize those original findings with respect to specific application scenarios and specific user groups. Systematic review studies deliver such overviews based on an exhaustive manual screening of available digital libraries, providing a qualitative analysis of included studies, and unbiased performance comparison of the corresponding CH solutions [17,18]. The examples of such review studies offering a useful insight into the spectra of the related wearable technologies, target user groups, and application domains are plentiful. Wilde et al [19] reviewed the usage of apps or wearables for monitoring physical activity and sedentary behavior and emphasized the barriers and facilitators for their adoption. A scoping review [20] summarized the practices and recommendations for designing, implementing, and evaluating mobile health (mHealth) technologies to support the management of chronic conditions of older adults, considering articles published from 2005 till 2015. Kvedar et al [10] focused on the concept of CH as an overarching structure for telemedicine and telehealth and provided examples of its value to professionals and patients. In the study by Liu et al [21], materials, design strategies, and powering systems applied in soft electronics were reviewed. It also summarizes the application of these devices in cardiology, dermatology, electrophysiology, and sweat diagnostics and discusses the possibilities for replacement of the corresponding traditional clinical tools.

The transformation of the wearable landscape in the last decade is thus evidenced in a plethora of original articles and patent documentation and summarized and compared in numerous focused systematic reviews [3,10-16,19-21]. In this paper, we scoped the wearable technology field over the decade, starting from 2010 to February 2019, to identify trends in literature with respect to 4 important pillars: technology, safety and security, prescriptive insight, and user concerns. The collected literature reflects on the achieved progress, open issues, perspectives, and gaps in the development of wearable systems for future CH domain. The covered topics mainly relate to enabling technology: sensing, data aggregation and processing, communication protocols, power supply, data protection, and data analytics. However, the results of numerous pilots and experience gained with consumer wearables provide an insight into different user-related concerns. After exploring the literature published over the last decade, we have summarized state-of-the-art technologies, future research focus, and paper statistics related to the following key issues: enabling technology topics, application of wearable sensors in CH, and different user concerns.

With the more general, high-level perspective on the research on wearable technology, user-related concerns and challenges experienced over broad application area, this scoping review aimed at overlooking research trends unconstrained to a particular user group, health condition, or lifestyle scenario and including both mHealth and smart living environments. The extensive search scope is supported by automated search procedures relying on natural language processing (NLP) algorithms. The trends over the last decade were analyzed using a set of identified articles from 3 large digital libraries.

### Purpose of This Review

Many studies elaborating on the use of sensors and wearables in assisted living environments, CH, and wellness and fitness apps were published in the last decade [3,10-16,19-22]. Those studies provide significant input for designing future CH systems, indicating benefits, but also shortcomings, barriers, and user feedback [19,23-29]. Nevertheless, there is a lack of studies with a general overview of the nature and extent of published research in that context.

This study aimed to identify and scope the scientific literature related to wearables in health monitoring, as measured by trends in the research evidence available in 3 large digital libraries: Institute of Electrical and Electronics Engineers (IEEE), PubMed, and Springer. The study scoped the field from several perspectives aiming to capture key drivers and major constraints in the deployment of wearable technology for health. The enabling technology relies on advances in sensing, processing, communications, and data protection. Conversely, multiple user perspectives imply privacy, utility, complexity, price, relevance, reliability, and significance of delivered feedback.

The objective of this study was to scope the research on wearable technology for health with regard to the following research questions:

- What are the most significant research trends and milestones on wearables seen as an enabling technology and as a key driver facilitating CH solutions?
- What are the most critical identified barriers and concerns from the technology and user perspectives and what trends are reflected in the research literature relating to these issues?

As an added value, this review can help identify the topics that need more detailed research in terms of elaboration of the obstacles and potential breakthroughs. The list of relevant articles resulting from this study can be filtered with respect to different fields (eg, keywords) to identify articles of interest for a systematic review in a specific subfield. The details in the list facilitate fast manual screening and selection of the subset of articles for further qualitative analysis. This type of preliminary search in planning a systematic review provides valuable answers on the feasibility (ie, does any evidence in literature exist), relevance (ie, has a similar systematic review already been done), and amount of time needed (ie, volume of the found evidence) to conduct a systematic review.

## Methods

### Scoping Review Methodology

This study adopted a scoping review methodology to identify and process the literature on wearables published from January 2010 to February 2019. Using a scoping technique, we aimed to examine the research evidence in the broad field of wearables, analyzing technology trends, including the resolved and emerging issues. The lack of a qualitative analysis of identified papers, the broad topic range, and the number of studies involved defined our approach as a scoping review and differentiated it from a systematic review [30,31]. The purpose of this study fully complies with the aims of a scoping review “to search, select and synthesize the knowledge addressing an exploratory question to map key concepts, types of evidence, and gaps in research,” as defined by Colquhoun et al [32]. Systematic reviews in the field of wearables, for its breadth and depth, have to focus more narrowly on wearable solutions and user concerns in a prespecified application scenario to facilitate qualitative analysis of included studies.

All emerging review types share their basis in scientific methodology, that is, they rely on formal and explicit methods for search and assessment of published studies and synthesizing of research evidence in conclusions on a well-defined research question [17]. One of the protocols for systematic reviews in health care, the Preferred Reporting Items for Systematic Review and Meta-Analysis (PRISMA) [18], provides a good example of thorough and rigorous checklist guidance. The corresponding PRISMA flow diagram illustrates the information flow reflecting the number of studies in different systematic review stages: study collection, study scanning, eligibility evaluation, thorough qualitative synthesis, and quantitative synthesis in meta-analysis [18]. The methodological framework for scoping reviews is underpinned by this exact and transparent way systematic reviews are conducted [17], providing sufficient details to reproduce the results. The workflow for a scoping review proposed by Arksey and O’Malley [30], and adopted in this study, includes 5 stages:

- Identification of a research question;
- Identification of relevant studies;
- Study selection;
- Charting the data; and
- Collating, summarizing, and reporting the results.

**Figure 1:**
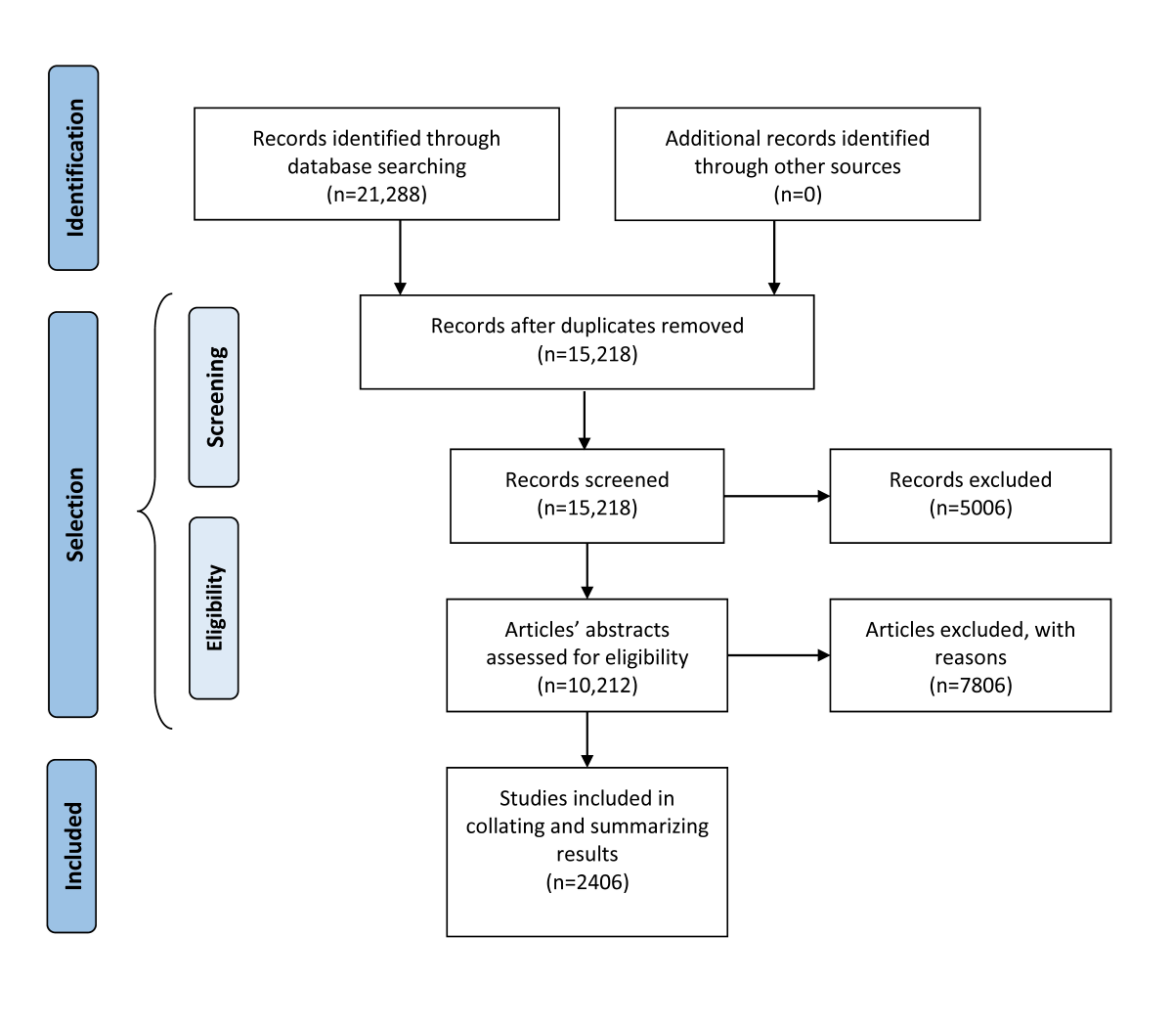
The review workflow reflecting the number of articles identified, screened, processed and removed in each step. The remaining articles were used for aggregating and summarizing the results.

The identification of relevant studies and study selection stages in the scoping review methodology corresponds to the PRISMA workflow phases: study collection, scanning, and eligibility evaluation. To ensure transparency, we have enclosed the workflow chart to illustrate the number of identified, scanned, and included articles in this scoping review (Figure 1).

The scope of this study was substantial and the collected research evidence on wearables surpassed the potentials of a manual search. Relying on the advances in NLP algorithms, the NLP tool kit [33] was used to ensure an efficient and exhaustive search of the literature corpus. The NLP tool kit is designed to automate the literature search, scanning, and eligibility assessment in the PRISMA methodological framework for systematic reviews [18], which are aligned with the scoping review phases: identification of relevant studies and study selection.

In the following sections, we clarify the usage of the NLP tool kit for study identification, selection (ie, scanning procedures and eligibility criteria assessment), and charting the data. It is worth noting that no quality assessment of the selected articles has been conducted, as this review has a scoping character. Instead, in the final step, we collated, summarized, and reported the results by aggregating the included studies to address the objectives of this review.

### Setting Up the Natural Language Processing Tool Kit

This stage concerns the development of a plan comprising decisions on which digital libraries will be queried, relevant time span, suitable keywords, and properties that should be satisfied. This scoping review has employed the NLP tool kit [33] enabling both automated search, scanning, and processing procedures. The NLP tool kit ensures compliance with the terms of use of the digital libraries, with regard to the number of requests per unit time.

The NLP tool kit input parameters are a collection of keywords that are used to identify potentially relevant articles and a set of properties that should be satisfied by the identified articles. The input is further expanded by proposing synonyms to the search keywords and properties. Synonyms can be provided by the user or proposed by the tool kit and fine-tuned if needed.

*Keywords* are search terms or phrases that are used to query a digital library (eg, “health” and “ambient and assisted living,” “health” and “enhanced living environment”). Eventual duplicates in the results are removed in a later phase. *Properties* are words or phrases that are being searched in the title, abstract or keywords section of the articles identified with the keywords. Examples of such properties employed in this study are “monitoring,” “recommendation,” and “detection.” *Property groups* are thematically, semantically, or otherwise grouped properties for a more comprehensive presentation of results. For example, the property group for the set of properties given in the example above can be “information delivery.” Table 1 summarizes the relevant input categories used in this work.

*Start year* indicates the starting year of publishing (inclusive) for the papers to be included in the study. *End year* is the last year of publishing (inclusive) to be considered in the study. This review encompasses studies published from January 2010 to February 2019. *Minimum of the relevant properties* is a number denoting the minimum number of properties that an article has to contain to be considered as relevant. In this study, this value was set to 3, providing a right balance between falsely identifying relevant papers and potentially missing a relevant paper.

When researches perform a scoping review according to the above-mentioned methodology, the actual tasks they perform involve searching digital libraries with different search phrases, often involving complex Boolean conditions. The NLP tool kit counterpart to these phrases are the keywords described above. By screening the title and abstract, a reviewer determines whether the article is indeed relevant for the study. In the NLP tool kit, this process is automated using the properties and their synonyms to define what we are looking for in an article. Articles that contain more properties are considered as more relevant. Undoubtedly, a human reader might better understand the context and better assess the relevance of an article. However, the NLP tool kit is mimicking these tasks, but in an automated and more thorough way, providing incredible efficiency of the scoping review process. For more information about the actual implementation, we refer the reader to the study by Zdravevski et al [33].

**Table 1:** The natural language processing tool kit input parameters: keywords, properties, and property groups.

Input parameters	Natural language processing tool kit input parameters
Keywords	health AND any of (ambient assisted living, ambient intelligence, assistive engineering, assistive technologies, enhanced life environment, enhanced living environment, enhanced support environment, hearables, home technologies, nearables, smart environment, smart home, wearables)
Property groups (properties)	Technology (cloud, communication, communicating, transmission, data processing, data analytics, battery, energy, fog, edge, protocol, sensor, sensing, detector) Information delivery (monitoring, recommendation, detection, supervise, catching, detecting, spotting) Concerns (acceptance, adoption, privacy, concealment, intrusiveness, intrusive, technology acceptance, technology adoption, seclusion, meddlesomeness) Safety and security (protection, reliability, dependability, safety, safe, security)

### Identification of Relevant Studies

Upon provision of the defined input categories, the literature search was started using only the specified keywords to query the selected digital libraries. The NLP tool kit indexed the following digital libraries (ie, sources): IEEE Xplore, Springer, and PubMed. It is worth noting that the NLP tool kit has used search engines of the corresponding publishers and retrieved the search results. Depending on the digital library in each search, the number of retrieved articles was constrained. In the PubMed library, all articles matching the given search criteria were retrieved for further analysis. The IEEE’s search engine limits the number of articles in each search to 2000, all of which were retrieved. For Springer, the search for each keyword separately is limited to 1000 articles or 50 pages with results, whichever comes first, sorted by relevance determined by Springer.

### Study Selection

After articles had been identified based on the specified keywords and retrieved from the publishers, the study selection (screening and eligibility assessment) procedures followed.

Upon merging the results from multiple independent keyword-based searches, some articles could be present multiple times because they could be identified by different keywords or in multiple libraries. Therefore, the collected articles were screened, and duplicates were removed using their digital object identifier (DOI). In addition, the screening process discarded articles that were not published in the required time span (ie, last 10 years) or for which the title or abstract could not be analyzed because of parsing errors, unavailability, or other reasons.

The selection of studies from the remaining subset of articles relied on the advanced functionalities enabled by NLP tools. The NLP tool kit automates analysis of a title and abstract for each study, significantly reducing the number of articles for manual screening. The automated eligibility analysis involved the following processing: tokenization of sentences [34,35] and English stop words removal, stemming, and lemmatization [35] using the Natural Language Tool kit library [36]. Stemmed and lemmatized properties were searched in the cleaned abstracts and titles, and each article was tagged with the properties it contained.

The processed articles were selected (ie, labeled as relevant) if they contained at least 3 of the predefined properties in its title or abstract (considering the above NLP-enhanced searching capabilities, thus performing a rough screening). To help in the eligibility analysis, the selected articles were sorted by the number of identified property groups, number of identified properties, number of citations (if available), and year of publication, all in descending order. For the relevant articles, the tool kit automatically generated a bibliographic file (as defined by BibTeX reference management software) for simplified citations.

The information flow diagram illustrating the numbers of identified, screened, processed, and removed studies in the automated NLP procedure is presented in the Results section (Figure 1) to ensure transparency and reproducibility.

The listing of the relevant identified articles extracted from IEEE, PubMed, and Springer is available in Multimedia Appendix 1 as an Excel file with the following fields: DOI, link, title, authors, publication date, publication year, number of citations, abstract, keyword, source, publication title, affiliations, number of different affiliations, countries, number of different countries, number of authors, BibTeX cite key, number of found property groups, and number of found properties. These additional files facilitate refined manual search of the articles with specific filtering criteria. The subset of targeted articles can subsequently be retrieved from their publisher and manually analyzed for potential inclusion in the qualitative and quantitative synthesis. It should be noted that not all the references provided within this study are from the identified set of relevant papers. Some additional papers identified in a manual search were used to illustrate and confirm the findings of this scoping review. However, these referenced papers from other libraries have not been used to identify trends in this scoping review.

To replicate the results manually, the keywords in Table 1 have to be used to inquire the selected digital libraries using their search engines. The properties serve for identification of the relevant articles by scanning titles and abstracts of the identified studies. The results can be compared with the resulting list of included studies, provided in Multimedia Appendix 1.

### Charting the Data

To answer the research questions, we defined indicators to be extracted from the selected studies. The trends in the past decade were analyzed relying on a broad scope of literature. The processed and retained relevant articles were aggregated by several criteria:

- Source (digital library) and relevance selection criteria;
- Publication year;
- Digital library and publication year;
- Search keyword and digital library;
- Search keyword and year;
- Property group and year;
- Property and year, generating separate charts for each property group; and
- Number of countries, number of distinct affiliations and authors, aiming to simplify the identification of collaboration patterns (eg, written by multiple authors with different affiliations).

These aggregated metrics are available in the form of comma-separated values files and charts. The plotting of the aggregate results was integrated and streamlined using the Matplotlib library [37] and NetworkX [38]. The NLP tool kit enables graphical visualization of the results, where each node represents one of the properties, each edge connects 2 different properties, and its weight is determined by the number of articles containing both properties connected by that edge. Articles that do not contain at least 2 properties, and properties that were not present in at least 2 articles were excluded. For a clearer visualization, only the top 25% property pairs by the number of occurrences are shown in a graph.

A similar graph for the countries of affiliations was generated. The top 50 countries by the number of collaborations were considered for this graph. Countries and an edge between them are shown if the number of bilateral or multilateral collaborations was in the top 10% (above 90th percentile) within those 50 countries.

### Collating, Summarizing, and Reporting Results

Using charted data and extracted evidence, we were able to analyze the trends in data and provide qualitative analysis for each thematic segment (as defined by the property groups). The results were reported with regard to the raised research questions. The meaning of these findings was related to the study purpose, and the potential impact on the future research direction was discussed.

## Results

### Number and Distribution of Identified Articles

**Figure 2:**
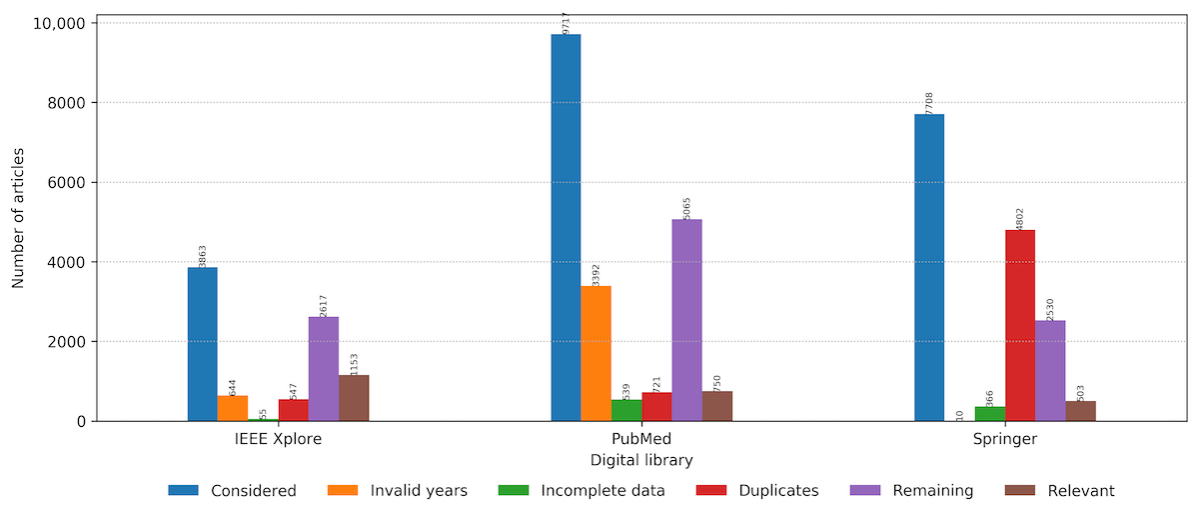
Statistics on the number of articles in the identification and study selection (screening and eligibility assessment) phase for each digital library. IEEE: Institute of Electrical and Electronics Engineers.

Using the NLP tool kit and searching 3 digital libraries: PubMed, IEEE, and Springer, we identified 21,288 studies with potential relevance (Figure 1). Duplicates that emerged in multiple independent searches were removed, reducing the total number to 15,218 studies. The first screening process further eliminated 5006 studies published before 2010 or for which the title or abstract could not be analyzed because of parsing errors, unavailability, or any other reason. The remaining 10,212 studies underwent an automated eligibility assessment using the advanced NLP tool kit functionalities. After processing, the articles were tagged with identified properties, and all articles containing less than 3 properties were removed. Overall, 2406 articles were deemed eligible for further manual inspection and inclusion in identifying the research trends and summarizing the results. The statistics on the number of the collected articles, duplicates, articles with invalid time span or the articles with incomplete data, and relevant articles are presented in Figure 2 for each digital library.

**Figure 3:**
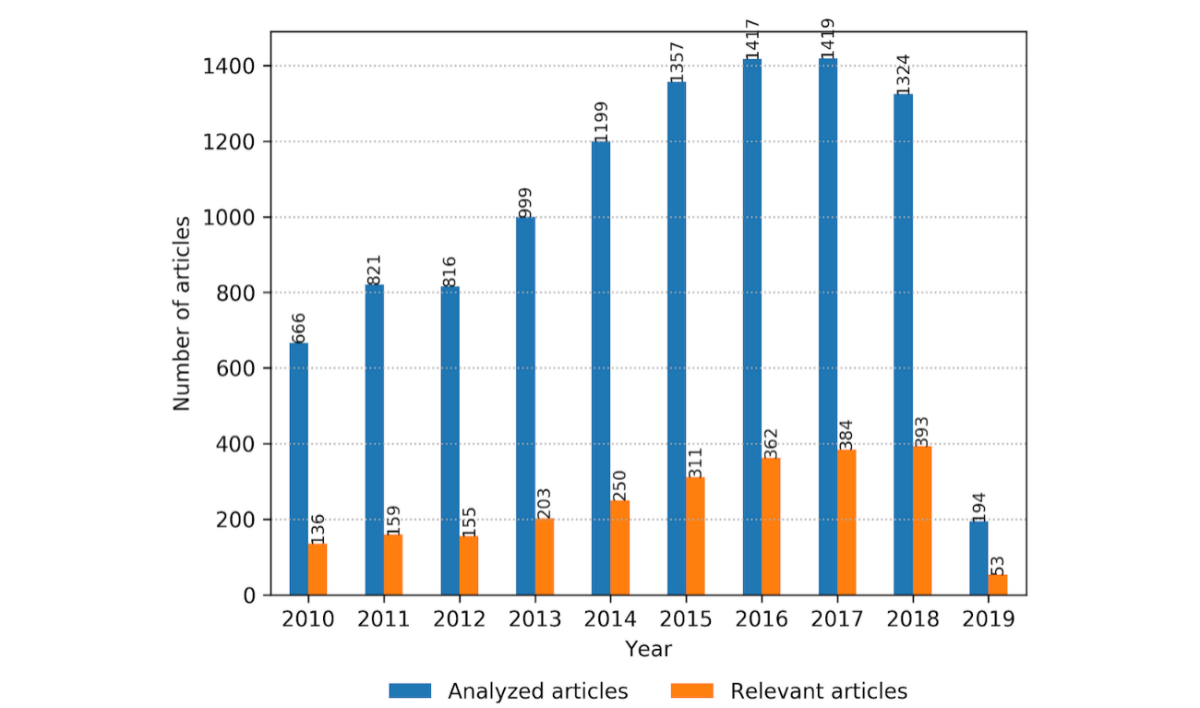
The number of analyzed articles versus the number of selected relevant articles on wearable technologies per year from January 2010 to February 2019.

The distribution of the number of collected and relevant articles per year is presented in Figure 3. An increasing trend in the number of collected articles can be noticed from January 2010 to February 2019. The same trend is followed by the number of included articles, which rises from 136 in 2010 to 393 in 2018.

**Figure 4:**
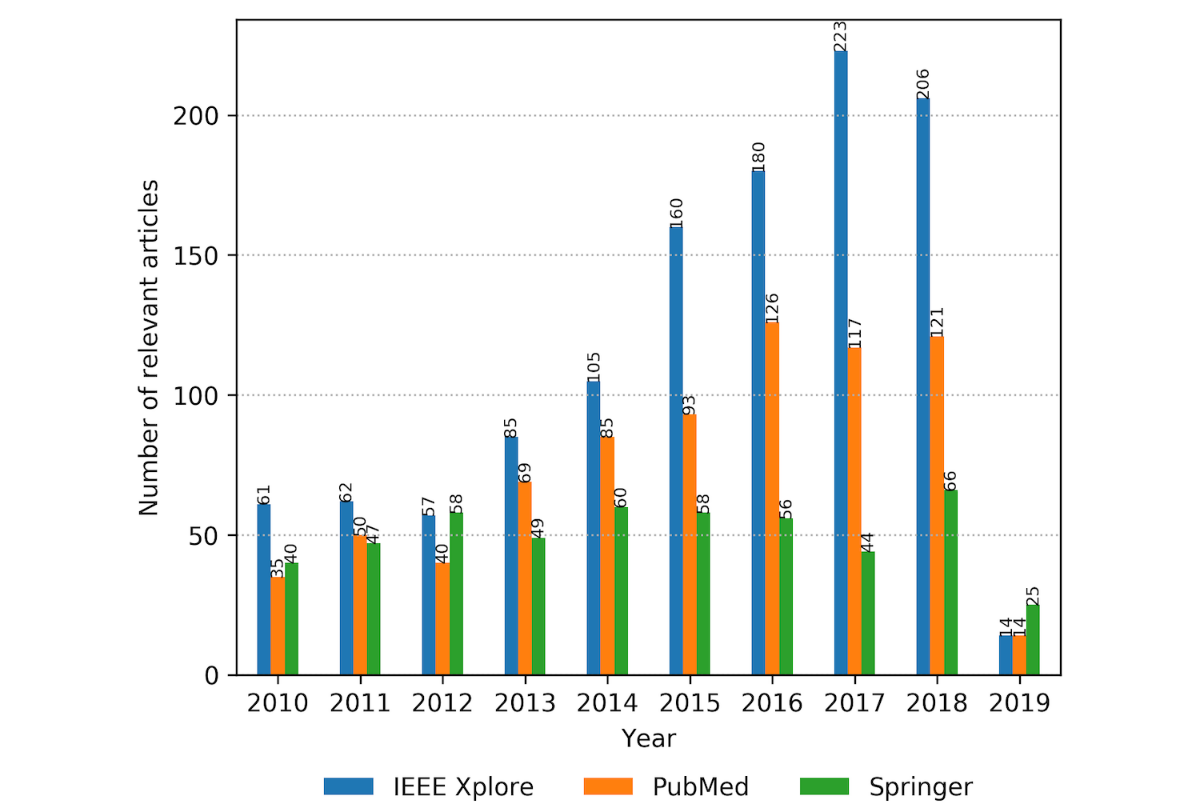
The number of identified relevant articles per year from January 2010 to February 2019, sorted by the originated digital library (Institute of Electrical and Electronics Engineers [IEEE], PubMed, and Springer).

Combining the information on the digital library (source) and publication year of the identified relevant articles, the obtained distribution reveals that IEEE, being a more technology-oriented library, has an increasing trend in the number of relevant articles from 2010, peaking in 2017 (Figure 4). PubMed leads in the number of articles dealing with CH and assisted living and covers more of the searched properties related to user concerns. The number of PubMed articles follows an increasing trend from 2010 and saturates in research evidence from 2016 onward. The Springer library shows an oscillating trend from 2010 to February 2019, with an average of around 50 articles per year.

### Geographical Distribution and Collaboration Evidence

**Figure 5:**
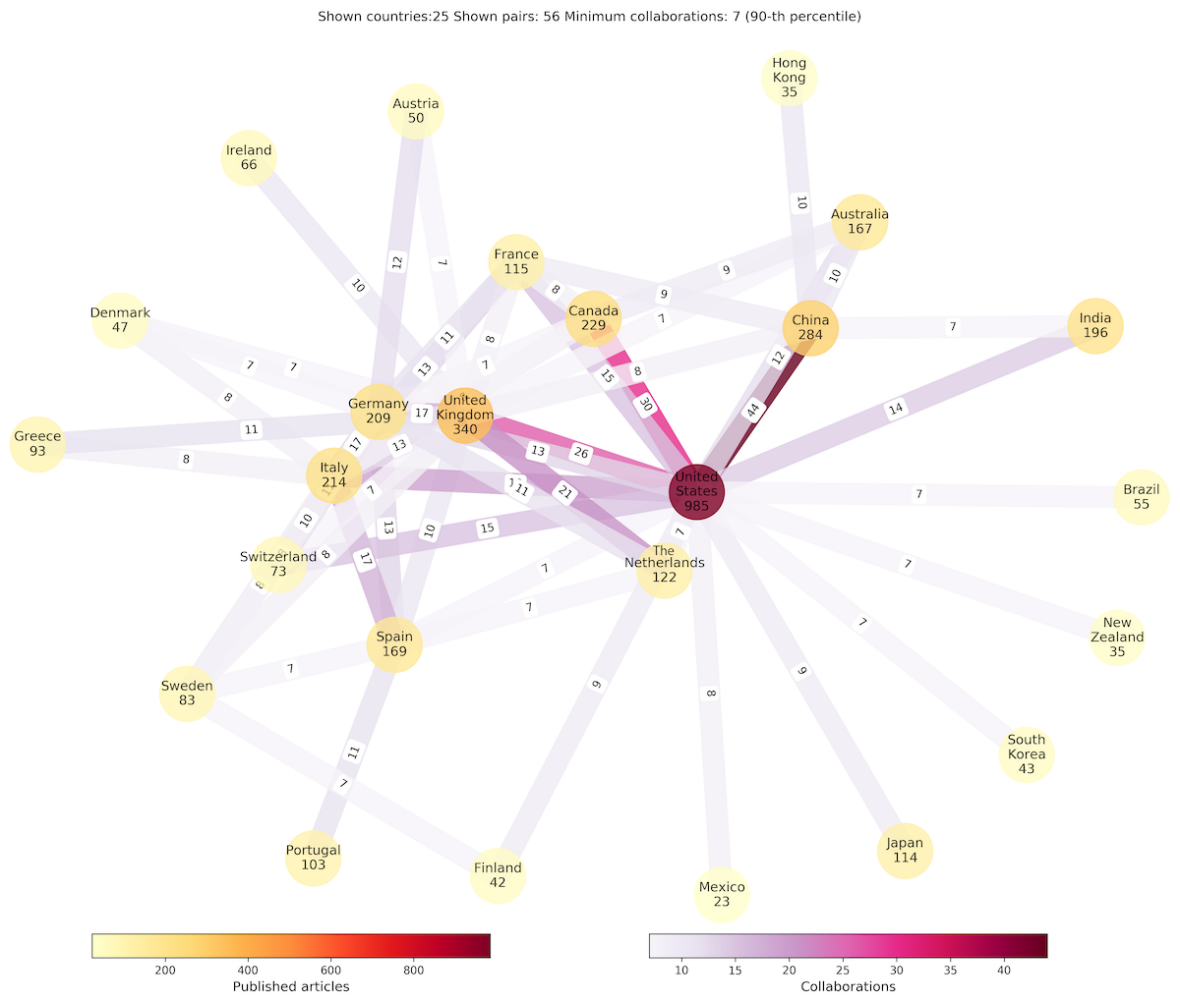
The number of relevant research papers per country and collaboration links with the annotated number of joint articles. For clarity, only 25 countries (nodes) with significant research contribution and minimum 7 joint collaborations (edges) are presented.

The authors’ affiliations were used to identify wearables’ research community clusters and eventual hubs at the research forefront. Multiple country associations were discovered, but for the sake of presentation clarity, the graph in Figure 5 shows 25 countries (nodes) and 56 edges with at least 7 joint articles (90th percentile) specified as edge weights. The number of papers per presented node is color coded, where violet corresponds to the higher and yellowish (paler) color to the lower number of articles. The identified hubs, United States, Canada, United Kingdom, Germany, China, and Italy, feature both national and international scientific production, whereas the strongest edges exist between the United States and Canada and between the United States and China. The collaboration patterns largely correspond to the neighboring geographical areas. The European countries demonstrate active collaboration scheme as well. The United States, United Kingdom, and China have significant national scientific production in the analyzed research domains.

### Keywords Statistics

**Figure 6:**
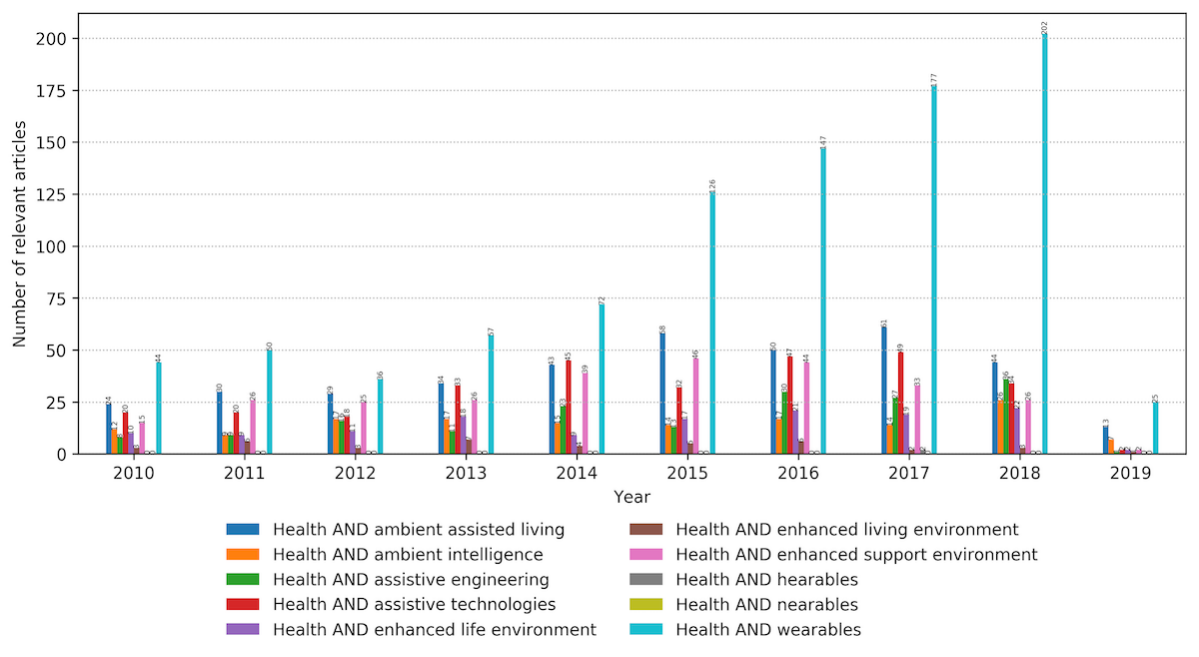
Distribution of the number of relevant articles with each of the defined keywords on an annual basis from January 2010 to February 2019.

**Figure 7:**
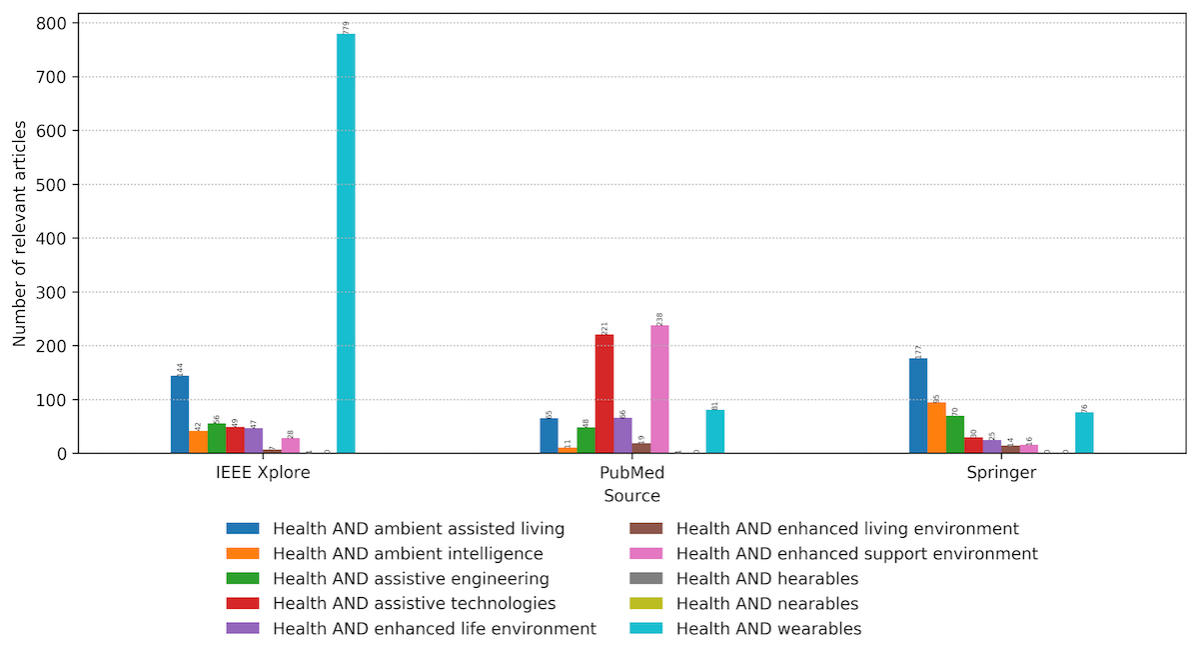
The number of relevant articles containing each one of the keywords per digital library. The data are aggregated within the defined period. IEEE: Institute of Electrical and Electronics Engineers.

The selected keywords used to map the literature corpus on wearables with respect to the set research questions appear in the relevant articles with different distributions. Figure 6 presents the annual number of research papers identified by the search engines of 3 libraries with the defined keywords and additionally filtered manually based on their relevance to the defined properties. Please note that the internals of their search engines are not known, and the libraries might differ in the way they look for these keywords: only in a title, keywords section, abstract, or a whole article. Depending on the digital library, the ratio of the relevant papers containing specific keywords changes (Figure 7). The IEEE digital library has a focus on enabling technology for CH, in terms of novelties in wearable sensing, data processing analytics, computing, and communication protocols. PubMed publications are also oriented toward CH technologies from an assistive and supportive perspective. Springer publications cover slightly different topics, focusing mainly on ambient assisted living (AAL) and ambient intelligence and generally contain more technical articles that address assistive technologies.

### Statistics of Properties

**Figure 8:**
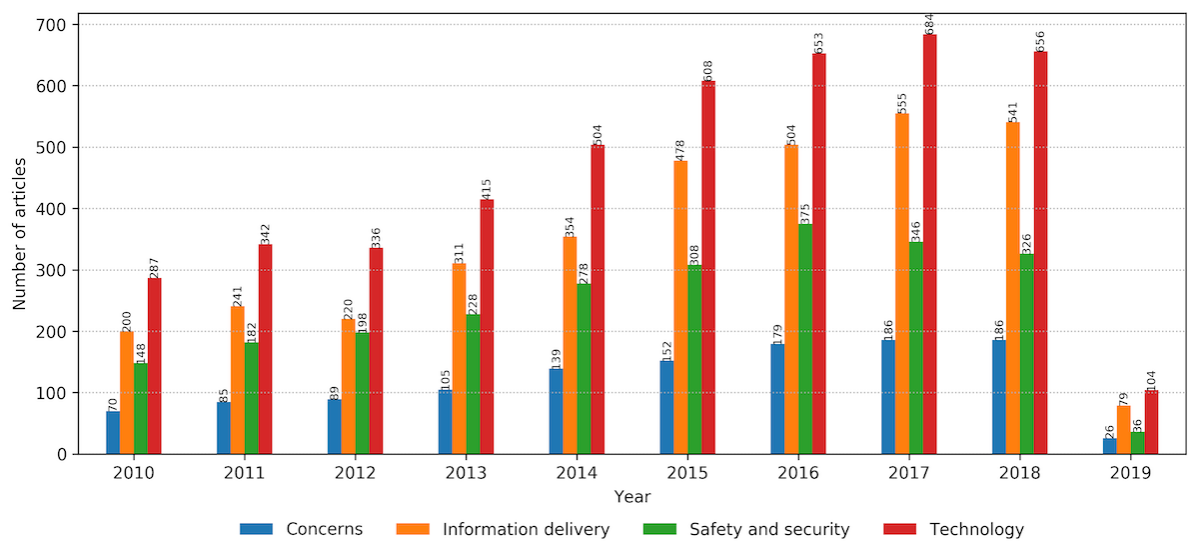
The number of relevant articles related to each property group per year within the predefined time frame from January 2010 to February 2019.

**Figure 9:**
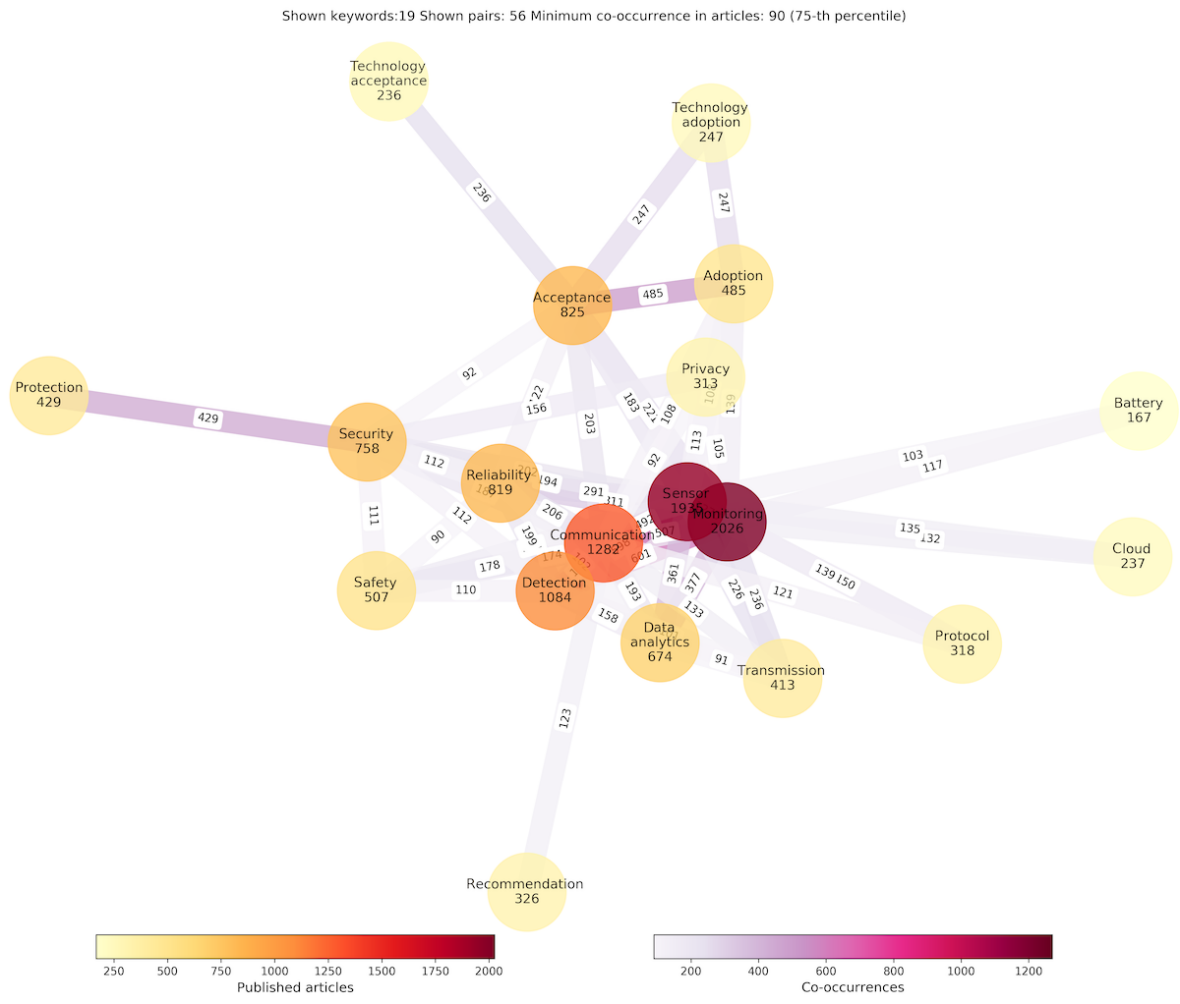
The property graph indicates the number of relevant articles with each property and the co-occurrences of properties in those.

As the number of research articles increases within the observed time frame, the number of articles dealing with associated topics summarized in property groups increases accordingly (Figure 8). The increasing trend is accompanied by the stable ratio of papers, with technology-related publications being the leading in number, followed by research related to information delivery, safety and security, and user concerns. When the view is zoomed from property groups to properties, the graph reveals the centrality of *monitoring* as the essential function of a wearable system tightly connected with the key technology: *sensing* (Figure 9). The 2 properties interrelate with communication, detection, reliability, safety, security, transmission, data analytics, and privacy as technologically empowered concepts. Acceptance is the key user-related property in the graph core, with privacy and protection to follow.

## Discussion

### Principal Findings

Wearable medical devices play a critical role as an enabling technology and as a key driver that has facilitated the emergence of CH solutions. This paper presents an overview of the most important milestones and trends that have driven research and development initiatives on wearable technology domains in the last decade. Simultaneously, it aimed to identify the most critical barriers or concerns, as far as technology and user aspects are concerned, that hinder the generalized adoption of wearables and still require further research.

The adopted methodology used the NLP tool kit for searching in 3 digital libraries, PubMed, IEEE, and Springer, for papers that address research on wearable technologies for medical applications. In the following, we address the findings related to the research trends in technology, information delivery, user concerns, safety, and security.

### Technology as a Key Driver

**Figure 10:**
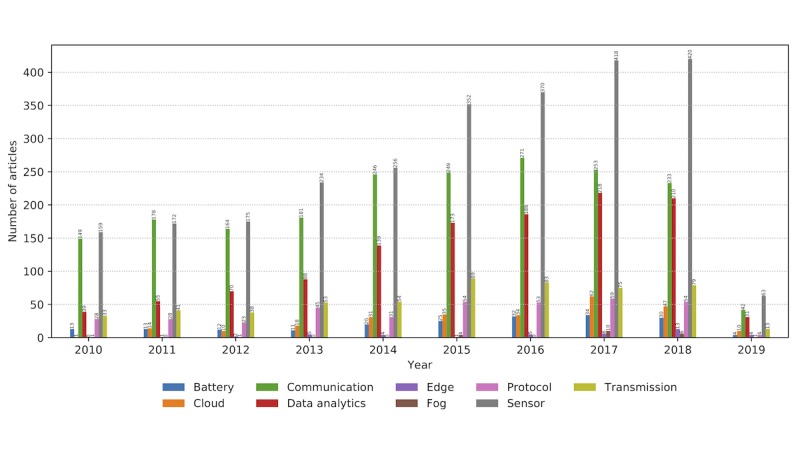
The number of relevant articles containing each property grouped primarily into the technological domain in the period from January 2010 to February 2019.

The literature (Figure 10) reflects the intense research and development in sensor design, communication protocols, and data processing and analytics. The emergence and evolution of concepts of edge computing, cloud, and fog could be easily tracked. As technology is a key enabler of future CH systems, we briefly review significant technological advances in the comprising components of a wearable system.

#### Evolution of Sensing Technology

Available sensors and their characteristics largely influence the design of CH systems. The direct sensor’s contact with the body implies their stiffness and size, as the most important features concerning comfort and measurement accuracy. The placement of wearable sensors influences their characteristics, user acceptance, and engineering requirements. As sensors evolve from wearable and implantable to ingestible sensors, barriers arise on multiple pathways: regulatory, technical, and translational [39].

The marked progress in wearable sensors is linked to advances in material science and embedded systems. Smart garments or electronic textiles, featuring sensor flexibility, made the first promise toward seamless and pervasive monitoring. The sensor integration into fabrics varies from *garment level,* assuming sensor integration at a later stage, to *fabric level* implying sensor integration by application of coatings to the fabrics [40]. The striving level is a *fiber level* [40] implying integration of conductive threads and fibers in the knitting process to result in a *smart fabric* (a concept first proposed about 20 years ago [41]).

Microcontroller-based systems can as well be included within different textile fabric for health applications [42]. Some products have already been approved and introduced to the market, but most of them are at a prototyping stage. The limitations arise at the electronic and textile integration step, slowing down technology transfer. In addition, there are multiple regulatory concerns, such as safety, reliability, and recycling [43]. Another promising technology for wearable CH solutions is microfluidics. Both sensing and drug delivery can be realized by combining microfabrication and liquid manipulation techniques with conductive elements on stretchable and flexible materials [44,45].

Low-power microelectronics, biocompatible materials, micro- and nano-fabrication, advances in data transmission, and management of sensor drift have driven the development of implantable biosensors [46]. Recent advances report the use of polyamide, flexible material for sensor platforms [47,48]. Research on flexible mechanical and electrical sensing has demonstrated great potential in in vitro diagnostics [49] and advanced therapy delivery [50]. Polymer-based switching matrices used for electronic skin to enable pressure sensing (robots, displays, and prosthetics), evolved into skin-attachable wearable electronic devices [48]. Another use-case involves surgical procedures, where these matrices are used in surgical procedures as part of mapping systems attached to the surface of the organs [50]. Active research directions in polymer sensors are focused on transparency [51], self-powering [52], and self-healing [53] capabilities.

The new generation of implantable sensing solutions for tissue and organ monitoring is enabled by advances in epidermal electronics based on soft lithography and thin-film sensors [46,54]. For example, electrocardiogram, blood glucose, and blood pressure sensors integrated with microstructures provide optical, thermal, and electrical stimulation [55].

Hearables are one of the latest wearable devices aiming to integrate sensing of multiple physiological signals into a single device [56]. The *in-ear* placement of such a device requires a flexible and comfortable fit and provides stable position regardless of the subject’s gross movements. The viscoelastic foam used as a substrate additionally ensures artefacts absorption, as the ear channel is affected by small movements, when speaking, swallowing, or chewing. The solution proposed by Goverdovsky et al [56] offers continuous measurements of cardiac, brain, and respiratory functions.

Implantable pacemakers, pressure sensors, cochlear implants, drug infusion pumps, and stimulators are all examples of implantable devices delivering therapy or providing physiological monitoring [39]. The majority of implantable devices currently operate in an open loop. New research challenges are focused on combining monitoring and therapy delivery for the optimized closed-loop personalized therapy [39]. The neural signal recording is ultimately the most demanding task, as it requires precise, low-power, and low-noise electronics and miniaturized and light weight implantable designs [57]. Neural implants face the hardest challenges in the translational pathway of the research-grade solutions into clinically approved products.

Ingestible sensors for image and data recording in gastrointestinal endoscopy have already proven their benefits in early detection of gastrointestinal cancers [58]. Ingestible, similarly to implantable devices, face challenges that shape the ongoing research: operation frequency selection, amplifiers, antenna design and performance, wireless channel modeling, increasing data rates, and power considerations.

Besides tracking basic physiological parameters (electrocardiogram, blood pressure, blood oxygen saturation, temperature, etc) sensing functions in wearable medical devices have also moved off the body toward contactless or seamless ambient embedded physiological sensing in, for example, keyboards, joysticks, steering wheels, bicycle handles, doors [59], mattresses [60], beds [61], and toilet seats [62]. The combination of such monitoring products with the data-driven services has promoted the development of the AAL concept. The AAL is a new ambient intelligence paradigm where new technologies are associated with the social environment, to transparently improve and assist the daily quality of peoples’ lives. Despite the high number of research and industry organizations already active in the AAL field, significant efforts are still needed to bring these technologies into a real-world usage [15].

#### Powering Wearables: Constraining Consumption and Energy Harvesting

One of the limitations for a widespread adherence to wearable electronic products concerns the power supply needs [7,9,63]. Active wearable systems need to be comfortable, light, user-friendly, and power efficient. The identified research trends reveal that research on battery technology lags compared with research on other wearable system components (Figure 10). This implies that energy efficacy and efficiency remain an important design concern, both for wearable systems and in the design of networks to serve future landscape of wearables (notably fifth generation [5G] architectures).

Energy harvesting technologies have been explored as an alternative energy source to recharge power batteries or super capacitors. The ongoing research in this domain has investigated technologies to explore motion [64,65], thermal [66,67], optical, electromagnetic [68], solar [69], and chemical forms of energy [70]. However, miniature devices that can harvest proper levels of energy are still in their infancy.

Complementary efforts are being invested in the integration of power-efficient technologies and design techniques in wearable systems. Among those are energy-efficient and low-power wireless communication, voltage scaling, low-leakage and low-voltage complementary metal oxide semiconductors [71], and power-performance management.

#### Communication Protocols for Wearable Systems

The medical data are low in volume, but with strict requirements in terms of latency, link reliability, and security [7]. Wearable body sensor networks or BANs refer to sensor networks applied for acquisition or monitoring of vital physiological body parameters unobtrusively. These systems can be used in clinical settings or at home by patients or even healthy people who want to improve or monitor their health conditions.

BANs enable wireless communication in and around a human body in 3 different tiers: intra-BAN, inter-BAN, and the beyond-BAN. Intra-BAN communications refer to communications between on-body sensors, within the surrounding body area, enabling wireless data transmission to a personal server. According to the application and design parameters, the intranetwork can be wired or wireless, or even use the human body as a communication medium. Wired networks, as a second type of communication infrastructure for BAN applications, provide high-speed, reliable, and low-power solutions [72].

The international IEEE 802.15.6 standard enables delivering of low power, short range (in the vicinity or inside, within the human body) reliable wireless communications, with data rates from 75.9 kbps to 15.6 Mbps, making use of industrial, scientific, and medical bands, as well as frequency bands approved by national medical and regulatory authorities [73].

The inter-BAN communications include communicating data from personal devices such as smartphones to the access points, either in an infrastructure-based manner or in an ad hoc manner. Wireless BANs can interact with other existing wireless technologies such as ZigBee, wireless local area networks (WLAN), Bluetooth, wireless personal area network, video surveillance systems, and cellular networks [73].

Finally, the beyond-BAN tier connects the access points to the internet and other networks. Beyond-BAN architectures can be implemented in cloud or fog network infrastructures [74] implying *protocols*, *cloud-based systems,* and *fog systems* as research topics in the wearable CH domain. The major challenges in BAN are associated with media (path loss because of the body absorption), physical layer (minimization of power consumption with uncompromised reliability and interference), medium-access control layer (supporting multiple BANs in parallel application), security, and transmission (loss and delay sensitive real-time transmission) [75].

Limited spectra and the need for higher data rates drive the communication community toward the new generation of cellular networks such as 5G [22,63]. The high-speed data and low-latency features of 5G networks will allow wearable devices to communicate faster (in less than 1 millisecond) and perform real-time control. 5G will be a platform for various services and applications, with support to different communication requirements. The transition to millimeter wave (mmW) frequencies will require new communication architectures to be designed for specific mmW propagation. For protection and regulation of exposures to such frequencies, more appropriate metrics are needed, such as temperature elevation of the contact area [7].

The design of wearable antennas, with safety concerns, device-centric architectures, and smart device communication are some of the changes 5G will require. The development of 5G brought the promises supporting the wearables market, such as radio-frequency sensor charging [63], reduction in latency, high data rates and capacity, and network densification, enabling the massive number of deployed wearables per micro- or picocell [22].

The 5G architectures proposed to serve wearables include microbase stations for blanket coverage, whereas local coverage and data throughput should be ensured with small base stations and remote radio headers (RRHs) [7]. RRHs can also support different wireless technologies to ensure backward compatibility (Bluetooth, visible light communication (VLC), etc). The connection to cloud data servers via base stations enables storage, retrieval, and analytics of user-specific data. Realization of communications between wearables and network edge nodes can be done using licensed or unlicensed communication bands. Licensed communication bands provide quality of service at an increased cost at several levels: a service provider cost for more expensive licensed chips and more power consumed on licensed communication protocols. Unlicensed communication (eg, Bluetooth, WLAN, and VLC) is a cheaper, power-preserving option but limited in range [7].

#### Data Processing and Analytics

The large volume and heterogeneous data types collected using wearable technology have grown beyond the abilities of commonly used data processing techniques [76]. The necessity for reducing the volumes of captured data at the source, to reduce the power consumption and latency, brought processing closer to the sensor nodes, mapping the data algorithms to ultralow-power microcontrollers [46]. Preprocessing approaches, such as noise filters, peak detection, and feature extraction, allow for significant data reduction at the source [77]. Conversely, advanced data analytics imply sensor data integration, thus relying on the powerful devices located in the cloud. 5G should offer mobile edge computing to reduce latency and traffic demands to the central node. In the wearable scenario, communication between various user devices is fostered by 5G machine-to-machine communications, enabling local processing, low latency, and power saving [7].

High-performance computing permits efficient processing of large data volumes through a map-reduce framework [78]. Advanced data caching and in-memory processing coupled with GPU accelerators and coprocessors support intensive parallel operations. The availability of higher computational power enabled the rebirth of computationally intensive deep neural networks, resulting in superhuman performance and cutting-edge research in multiple domains. These are enabling technologies that will bring to reality the third generation of pervasive sensing platforms [46] that will integrate and extract information from a variety of sources: sensed data, clinical records, genomics, proteomics, and social networks, leading to a system-level approach to human health [79].

### Information Delivery and Valuable Feedback

**Figure 11:**
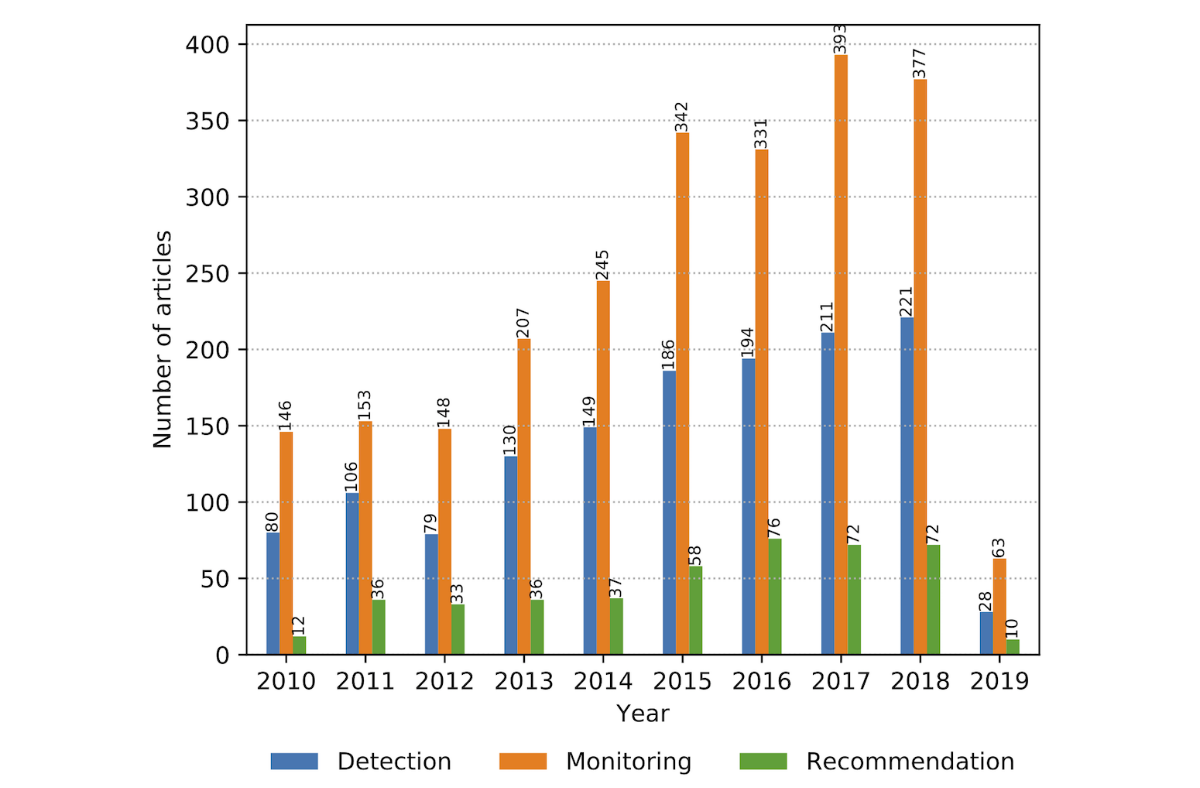
The number of relevant articles related to recommendations and feedback on health monitoring solutions from January 2010 to February 2019.

The research in the user-associated information delivery is primarily concerned with recommendations, provision of feedback, and real-time user insight (Figure 11). Current commercial wearable technologies, tracking vital signs and patterns of activity, lack the relevance for many potential consumers, presenting an additional burden [7]. The motivation to buy and use wearable systems has to be justified in a functional CH application context. The clear user benefit comes from a validated system that would transform collected data into manageable and useful information for medical action, safety instructions, or self-performance estimation and improvement.

To gain wider consumer preference, the information generated by wearables has to be fitted into specific contexts, offering the needed insight and recommendation on actions that should be taken. The second generation of wearable systems, which aims to enable context sensing, needs to integrate many different types of context information, such as sensor information, user profiles and preferences, activity patterns, medical history, and spatial information (location and environment conditions). If not strictly depending on medical condition, the timing, content, and frequency of prompting have to be adjusted to user preferences [80]. As a basic example, the time of day or night implies different content and presentation of the prompting messages because of the different level of user’s readiness and wakefulness [81]. The fusion of physiological and context sensing data will rely on sophisticated data analytics for extraction of relevant information and decision making on an action to be prescribed or advised to the user. The feedback to prompting messages generated in day-to-day system’s interactions with a user would ensure the adaptation to user preferences in time, relying on reinforcement learning.

The transformation of wearables from measurement devices into resources of reliable real-time information, history mining, and smart and personalized decisions would qualify them for health and performance monitoring solutions.

### Concerns

Wearables will reshape individuals and society, promoting self-care and health management, moving care outside hospitals, affecting enterprises, and revolutionizing health care [8,82,83]. Their seamless integration into consumers’ electronics is well witnessed throughout the Consumer Intelligence Series on wearables from 2014 and 2016 [8,83]. According to these sources, numerous user concerns such as design, accuracy, reliability, security, privacy, and dampened human interaction are becoming less worrying to the users. Research on sensor materials and communication solutions can provide advances in human-centered design and enhance the user experience.

Another big hurdle for deploying wearable systems in the real-world concerns technology acceptance [84,85]. Even though wearables are adopted by the millennials, the older population is still uncomfortable with using and relying on technology. As opposed to the smartphone, the use of wearables in fitness and well-being scenarios does not have clear usage need and benefits. Consumers complain about uncomfortable and unattractive design, short battery life, and frequent connectivity challenges [8]. With the first wearable devices, we have witnessed a *wearables fatigue* attitude, which is noticeable in a significant percentage of wearables being discarded within the first 6 months of use [86].

**Figure 12:**
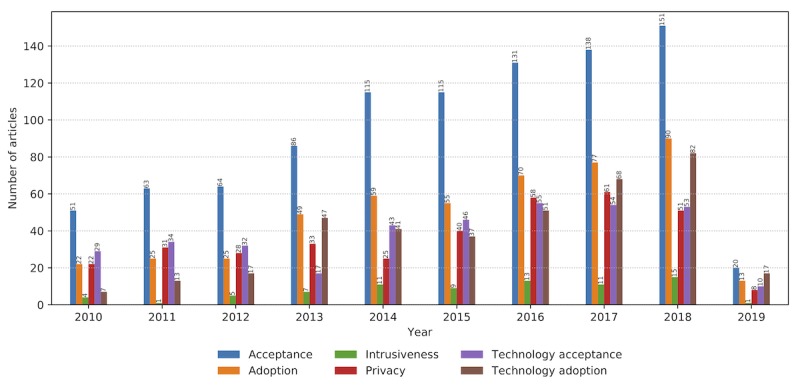
User-related properties and concerns addressed in the relevant literature corpus from January 2010 to February 2019.

Our findings are aligned with the outcomes of the user feedback reviews on wearable technologies [8,83], as the identified articles confirm the steady increase in research addressing user-related issues such as technology acceptance, technology adoption, and privacy (Figure 12). The primary design requirements are that a wearable device must be fit for the purpose and seamlessly adapted to the user’s lifestyle to be accepted.

Preserving privacy and confidentiality is a priority to be considered in design specifications. Communications should be encrypted and secured, and the involved parties should ensure confidentiality. This is particularly important in the case of wireless data communications that are easier to intercept [87]. Personal monitoring devices should unobtrusively authenticate the user identity using biometrics or key physiological signs (owner-aware devices).

Different user concerns, such as quality of experience, security, privacy, technology acceptance, and human-centered design, are relevant research topics in the wearable CH domain and can be used to identify future challenges and research trends. Although some of them (eg, quality of experience and human-centered design) might be decreased as the end user pool gains digital competence and technology matures with time, some of them (security, privacy, and technology acceptance) will probably evolve and mix with other, more societal research topics such as environmental impact, circular economy, and digitalization of society. These can raise a new set of concerns related to the socioeconomic impact of wearable technologies in combination with IoT and 5G technologies used for health care and lifestyle.

It is worth mentioning that another spectrum of concerns and barriers relates to the stakeholders involved in the provision and management of health care. Health professionals need scientific evidence on the reliability of collected data, the performance of analytical models mapping the collected data to disease progression, and eventually positive patient outcome in using wearable-based CH solutions [1]. Reshaping the health care critically depends on research work devoted to the design and evaluation of care pathways, provision of optimized feedback, and eventually providing evidence on long- and short-term cost-effectiveness of CH solutions [1,88].

### Safety and Security

**Figure 13:**
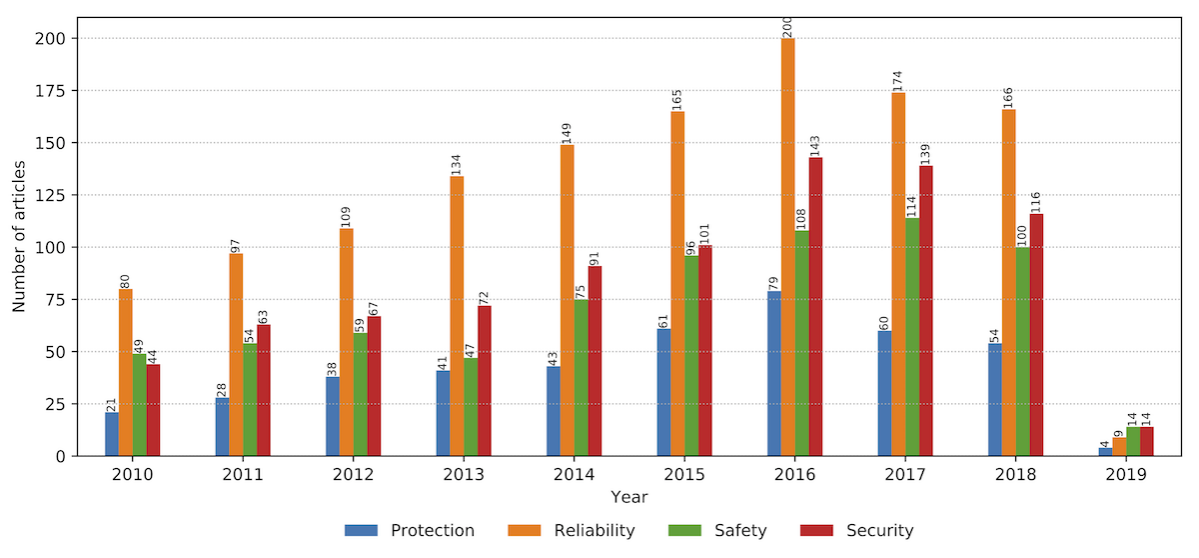
Safety- and security-related properties: protection, reliability, safety, and security. The trends in the period from January 2010 to February 2019 evidence their increasing presence (ie, relevance) as the wearable technology matures.

Safety and security are primary considerations for medical devices, tightly coupled with reliability at all system levels. Our findings confirm the increasing importance and research efforts related to these major user concerns (Figure 13). If the wearable device is required to perform safety-critical functions, the tolerance for error is zero. A failure in such a device can cost a life and that requires more effort and time (ultimately cost) to be invested in thoroughly testing and validating the device before it is deemed safe to use.

Along the life cycle of a wearable device, efficient mechanisms are required to detect and diagnose deviations occurring in the captured data. Correct differentiation of errors due to system-related faults from those due to a change in health status is a necessity. Increased level of false alarms (false positive) would prevent user reliance, reduce user alertness, and hamper user adherence to the provided feedback.

Both features, safety and security, are technology conditioned and should be ensured by the system design. Wearable medical devices are required to comply with IEC 62366-1:2015 standards [89] that regulate the application of usability engineering to medical devices to achieve approval. Two new EU regulations on medical devices issued in 2017 that will come into force in May 2020 are Regulation (EU) 2017/745 on medical devices [90] and Regulation (EU) 2017/746 on in vitro diagnostic medical devices [91]. These regulations will have a significant impact on the sector of medical devices that incorporates wearable technology. More stringent procedures for evaluation of medical devices and conformity should improve patient safety.

The CH paradigm involves more connectivity and communications into health care and medical devices. Any device connected to the internet is prone to be targeted for malicious purposes, putting it at a constant threat of damage, theft, and financial cost.

The number of detected data breaches in health care organizations has increased significantly in the last several years [92]. The reasons are not primarily technical but in part caused by the negligence and lack of knowledge of employees in treating this sensitive data and implementing the information security practices [92]. According to the 2017 Fourth Annual Data Breach Industry Forecast, health care organizations will be the most targeted sector with new, sophisticated attacks emerging. New security frameworks for mHealth are being proposed to ensure security and reliability of medical devices and personal health data [93-96].

After the General Data Protection Regulation was put in place on May 25, 2018, the requirements for data protection and privacy assurance have been raised and unified across Europe. The health care and monitoring systems have to adhere to the privacy by design principle, which requires the incorporation of privacy protection in systems design and not as an afterthought add-on solution.

### Limitations of the Study

This study considered only 3 digital libraries, and some relevant articles from nonindexed publishers were not considered. However, keeping in mind the size of the considered digital libraries, we believe that the obtained results are indicative for the purpose of the study.

All digital libraries that were used in this work have different internal search engines with different rules for the maximum number of papers that can be retrieved and different formatting of search results. The papers obtained for this study are the results of the same search query sent to those different search engines. However, keeping in mind the number of papers that were analyzed within this scoping review, we believe that specificities of the publishers’ search engines have limited impact and have not influenced the findings of this work.

In the future, the NLP tool kit needs to be extended to process more digital libraries. In addition, there is an apparent need of a Web app that will make it available to a wider audience. Until then, readers are encouraged to contact the authors if they are interested in using the tool kit.

### Conclusions

Wearable medical solutions, integrated into the wider concept of IoT, provide for pervasive data acquisition from a body and beyond, and rely on powerful data analytics, smart networking, and machine-to-machine communications to facilitate patient-centric, personalized, and holistic care. Although technological innovations and availability support the emergence of CH solutions, the widespread adoption of wearables is still hindered by numerous concerns related to reliability, security, and cost-effectiveness.

This scoping review maps the scientific literature related to wearable technology in health care starting from January 2010 to February 2019, identifying the research trends related to enabling technology, and the trends in addressing the concerns from both user and technology perspectives. The NLP tool kit supported search procedures applied over 3 large digital libraries, IEEE, PubMed, and Springer, which provided for a representative subset of 2406 articles on wearable technologies for medical applications.

On the basis of the investigated sample, the main findings reflect key drivers in the field, some research gaps and relevant topics that would benefit from more systematic qualitative knowledge synthesis:

- User concerns were the least addressed topic, whereas the enabling technology research was the main focus in the literature within the observed time period;
- Major breakthroughs were made in sensor technology, data analytics, communications, and computing architectures (edge and cloud);
- Research on battery technology and efficient solutions for energy harvesting has lagged, implying energy efficiency as one of the major constraints in designing wearable solutions for pervasive monitoring;
- Research on communication technologies focuses on 5G featuring low-latency, massive connectivity, and high capacity to mitigate the current challenges with respect to real-time feedback, energy, and computing constraints;
- The research related to the user-associated information delivery was mainly focused on monitoring and measurement information and much less on the provision of feedback recommendation and prescriptive insight; and
- The most addressed concerns from the user perspective were technology acceptance and issues related to safety and security, implying privacy and reliability as the most central topics.

This study confirms that applications of the wearable technology in the CH domain are becoming mature and established as a scientific domain. However, further research and development are required to improve their reliability, comfortability, and dependability levels. The research focus shifts from sensors and data analytics toward the sustainable delivery of valuable recommendations, reliable, energy-efficient, and low-latency communications and computation offloading. Sensor data integration goes beyond body-level integration to include context sensing, location and environment metrics, medical history, pattern of activities, and user preferences. This is essential for making wearables a robust patients’ representation interface and reliable node of the IoT infrastructure that makes CH a reality.

There is a further need to explore and provide the literature evidence supporting the positive experiences, improved patient outcomes, and cost-effectiveness of CH solutions. Practical adoption in the field still demands design and validation of new care pathways, optimization of interventional strategies, and a sound business model.

## Appendix

Multimedia Appendix 1 The list of the identified relevant research articles from the three selected digital libraries.

## Abbreviations

5G: fifth generation
AAL: ambient assisted living
BAN: body area network
CH: connected health
DOI: digital object identifier
IEEE: Institute of Electrical and Electronics Engineers
IoT: Internet of Things
mHealth: mobile health
mmW: millimeter wave
NLP: natural language processing
PRISMA: Preferred Reporting Items for Systematic Review and Meta-Analysis
RRH: remote radio header
VLC: visible light communication
WLAN: wireless local area network
